# Exopthalmos and Exopthalmia

**Published:** 1856-09

**Authors:** Sanford B. Hunt


### ART. III.—*Exopthalmos and Exopthalmia.*

By Sanford B. Hunt, M. D.

Both of these forms of disease are fortunately rare. My personal knowledge of them is too limited to afford any general conclusions, but having recently met a case of each variety their record may prove profitable.

### 1. *Exopthalmia of both Eyes.*

March 20, 1856. Mrs. G., Irish, aged about 46 years. A week since she had several teeth removed from the right jaw, which had been followed by swelling of the mouth. I found the gums of the affected side very much swollen, the tumefaction extending to the roof of the mouth and tonsillary region of the same side. A purulent fluid was oozing from the gums. I made free incisions in various directions through the swelling, through which considerable pus discharged. I saw her again on the two succeeding days when, considering her convalescent, I discontinued my attendance.

I was again called on the 27th. I found that the swelling in the mouth had subsided. She had, however, tonsillitis, and complained of intense pain running to the ear and through the orbits of the eyes. There was an erysipelatous redness extending beside the nose on the right side of the face.

On the succeeding day I found the eyelids tumid and œdematous, more upon the right than the left side. This condition increased until the entire conjunctiva of both lids and sclerotic was distended by fluid, so that the cornea appeared very much depressed, and, as it were, at the bottom of a pit. This distension was unaccompanied by any redness or injection of the vessels; it seemed a simple dropsical effusion. I made at first punctures, and afterward free incisions, in the conjunctiva, but the effusion was too gelatinous to flow.

The distension increased, and on the succeeding day the eyeballs were somewhat protruded. Accompanying this protrusion was more or less coma, which, as the exopthalmia increased, became more profound. She became blind, deaf and stupid, and died, after a profound coma of some two days' duration, on the 31st. Before death the protrusion was so great as to thrust forth the greater part of the eyeball from between the lids. The cornea maintained throughout a fair degree of brilliancy; the pupils were fixed.

The general symptoms preceding her death were apoplectic.

### 2. *Exopthalmia.*

Mr. M., three years and a-half since suffered from a destructive opthalmia, which entirely destroyed the left eye, and ruined the right for purposes of vision. Since that time he has been under the care of various oculists, in the hope of recovering the use of the right organ.

About March 1st he came under my care. I found at this time a congestive condition; the vessels of the eye being of a very dark color, and fully distended. Under the use of wine of opium this disappeared, and I was then enabled to judge of the condition of the cornea. Nearly the entire upper half was untouched by ulceration or opacity. On the lower half was a large opaque deposit of lymph, covering nearly one-third of the entire cornea. To the inner side of this was a perforating ulcer of considerable size, through which the iris protruded and the aqueous humor constantly discharged. The pupil of the eye could not at this time be recognized, but I afterward found it adherent to the inner surface of the opacity, in the outer lower part of the cornea.

Under the use of wine of opium the congested blue appearance subsided, and left a sub-acute inflammatory action, which I found materially benefited the condition of the cornea. A zone of vessels surrounded the perforating ulcer, and lymph was rapidly deposited upon its edges. Maintaining a stimulating treatment for some weeks, the ulcer finally closed entirely, and never opened again. During this time the opacity had very markedly diminished, and I was soon able to distinguish the pupil through it, as it became reduced in size and thinned. The cornea assumed its natural convexity, and it now only remained to bring the eye to a proper condition (as to inflammation) for the operation for artificial pupil. With a view to decreasing, as far as possible, the opacity, the inflammatory action was maintained so long as the absorbent action seemed going on. When it became evident that no further benefit was to be gained by the stimulants, they were discontinued, and the vapor of hydrocyanic acid applied to the corneal surface. Under this remedy the vascular condition of the cornea decreased, the vessels gradually shrinking. The eye was now ready for the operation for artificial pupil, but it was deferred on account of the heat of the weather.

During the early part of July a renewed inflammatory action was set up, which seemed for the time to be serious, but it yielded readily to the infusion of opium and lead.

July 23. I found a new inflammatory action occupying the inner angle of the eye. It did not look serious, but I discontinued the hydrocyanic acid and substituted the lead and opium collyium.

July 24. Was called at daylight. Found great chemosis and swelling of the conjunctiva, with intense orbital pain and general febrile reäction. Prescribed an active cathartic, and pencilled the eye with sol. nit. arg., dry cups to the temple, darkened room and light diet. During this and the succeeding day, the opthalmia seemed arrested. On the 26th, however, the bloodvessels assumed a livid appearance, the swelling of the conjunctiva was coupled with œdema of the lids and the region surrounding the orbit. The eye was projected somewhat beyond its natural position. From this time on, the intra-orbital swelling increased, pushing the eye out before it until the lids were widely separated, and the chasm between them filled with a gelatinous mass of swollen and injected conjunctiva, and circum-orbital tissue. New ulceration was set up in the cornea, and its outer laminæ entirely destroyed.

Prof. James P. White saw the case several times in consultation at this stage. There was an almost entire deficiency of secretion, and as all active measures seemed injurious, the treatment was at last confined to anodyne washes and poultices, by which the circum-orbital pain was somewhat relieved, and to general tonics.

Aug. 2. Swelling begins to subside.

Aug. 3. The tumor is considerably reduced. Contrary to my expectation the eyeball has not emptied itself through the ulcerated cornea. From this time the case has thus far gone on favorably, so far as the general health of the patient is concerned. His last hope of recovery from blindness is, however, irretrievably lost.

The first case described seems to have been an acute exopthalmos of anæmic character. It is not usual to witness so rapid a course of the disease, as it is commonly a chronic difficulty, and not generally fatal in its result. Mackenzie reports a case in which the exciting cause was cold, the progress and termination of which was entirely similar to that of Mrs. G. The fluid effused seemed to be gelatinous, and death probably occurred by an extension of this deposit to the brain. The treatment should doubtless be governed by the general health of the patient; but I am not aware of any form of exopthmalmos which could be benefited by depletion.

In the second case—that of exopthalmia—I find no parallel to any form of the disease recorded. It resembled somewhat exopthalmia fungosa, but the comparative ease with which the disease was controlled, as well as its rapid course, associate it somewhat with the phlegmonous variety.

I cannot, however, affirm that, aside from the conjunctivitis which existed, there was any inflammation deep-seated in the orbit. There was excessive engorgement, but at no time either local secretion or general febrile excitement. I am inclined to regard it as one of those viscous effusions described somewhat mistily by St. Yves, and mentioned by Mackenzie. The orbital pain was of that sort which indicated distension rather than inflammation.

In conclusion I have little to offer in the way of suggestion as to treatment. The accompanying ophthalmia should be treated as in other cases. In Mr. M.'s case the mustard pediluviam, sinapisms to the back of the neck, dry cups upon the temples, and general supporting treatment seem to have accomplished all that could be expected.

It is a matter worthy of remark that the fatty tissue of the orbit is so little liable to disease of any form. Its organization would seem to rank it as the seat of frequent phlegmonous attacks, while the rarity with which they occur is to well known to require mention.

